# The Mechanism of PMC (2,2,5,7,8-Pentamethyl-6-chromanol), a Sterically Hindered Phenol Antioxidant, in Rescuing Oxidized Low-Density-Lipoprotein-Induced Cytotoxicity in Human Retinal Pigment Epithelial Cells

**DOI:** 10.3390/antiox14080996

**Published:** 2025-08-14

**Authors:** Suman Chaudhary, Jean Moon, Zhengping Hu, Emil Kriukov, Sergio Pestun, Petr Y. Baranov, Yin-Shan Eric Ng, Patricia A. D’Amore

**Affiliations:** 1 Schepens Eye Research Institute of Massachusetts Eye and Ear, Boston, MA 02114, USA; schaudhary5@meei.harvard.edu (S.C.); jmoon13@meei.harvard.edu (J.M.); zhengping_hu@meei.harvard.edu (Z.H.); ekriukov@meei.harvard.edu (E.K.); sergio.pestun@sbs.org (S.P.); petr_baranov@meei.harvard.edu (P.Y.B.); 2 Department of Ophthalmology, Harvard Medical School, Boston, MA 02115, USA; 3 Department of Pathology, Harvard Medical School, Boston, MA 02115, USA

**Keywords:** geographic atrophy, retinal pigment epithelium, oxidized low-density lipoproteins, sterically hindered phenols, antioxidants, cytoprotection

## Abstract

Geographic atrophy or late-stage dry age-related macular degeneration (AMD) is characterized by drusen deposition and progressive retinal pigment epithelium (RPE) degeneration, leading to irreversible vision loss. The formation of drusen leads to dyshomeostasis, oxidative stress, and irreversible damage to the RPE. In this study, we used an in vitro model of oxidized low-density lipoprotein (ox-LDL)-induced human RPE damage/death to investigate the mechanism through which a sterically hindered phenol antioxidant compound, PMC (2,2,5,7,8-pentamethyl-6-chromanol), protects the RPE against ox-LDL-induced damage. We show that PMC exerts its protective effect by preventing the upregulation of stress-responsive heme oxygenase-1 (*HMOX1*/HO-1) and NAD(P)H: quinone oxidoreductase (NQO1) at the mRNA and protein levels. This effect was due to PMC’s blockade of ROS generation, which in turn blocked nuclear translocation of the nuclear factor erythroid 2-related factor 2 (Nrf2) transcription factor, ultimately preventing the upregulation of antioxidant response elements (AREs), including *HMOX1* and NQO1. The key role of HO-1 was demonstrated when the protective effect of PMC was inhibited by the knockdown of *HMOX1*. Additionally, PMC treatment under different experimental conditions and at different time points revealed that the continuous presence of PMC is required for the optimal protection against ox-LDL-induced cytotoxicity, defining the cellular pharmacokinetics of this molecule. Our data demonstrate the involvement of a key antioxidant pathway through which PMC mitigates the oxidative stress induced by ox-LDL and provides a potential therapeutic strategy for suppressing RPE degeneration/damage during AMD progression.

## 1. Introduction

Late-stage dry age-related macular degeneration (AMD), also known as geographic atrophy (GA), is a degenerative disease of the retina. It is characterized by slow and irreversible damage to the RPE, photoreceptors, and choriocapillaris, leading to central visual impairment [1,2]. In the retina, triglycerides and cholesterol are important sources of lipid metabolism. However, due to aging and genetic variants, lipid metabolism is dysregulated, which can contribute to the deposition of lipoproteins and lipid-rich drusen between the RPE and Bruch’s membrane [3,4]. Additionally, owing to the high-oxygen metabolic environment in the retina and the resulting significant generation of reactive oxygen species (ROS), the retina is highly susceptible to oxidative stress. This oxidative stress has often been linked with lipid peroxidation, endoplasmic reticulum stress, chronic inflammation, and DNA damage, which all contribute to the development of macular degeneration [5,6,7,8]. In particular, the oxidative stress and accumulation of oxidized lipids and lipoproteins exacerbated by dysregulated lipid metabolism have been linked with RPE dysfunction, degeneration, and death [4,9,10,11].

Typical antioxidant enzymes reduce ROS levels by scavenging free radicals, restoring the cellular redox status to promote the repair of cellular damage. However, excessive pro-oxidative conditions such as aging, cigarette smoke, a high-fat diet, and a lack of an antioxidant-rich diet can alter redox homeostasis, leading to retinal degeneration [12]. Previously, the multi-center, randomized controlled Age-Related Eye Disease Studies (AREDS) and AREDS 2 have indicated the efficacy of antioxidant supplementation in reducing the progression of dry AMD [13,14], which was recently confirmed by a follow-up post hoc analysis study [14,15], thus highlighting the importance of antioxidant compounds in reducing AMD pathology.

One of the primary pathways employed by the RPE cells to mitigate oxidative stress and maintain cellular homeostasis is the nuclear factor E2-related factor 2 (Nrf2) antioxidant response element (ARE) pathway. Upon oxidative stress, the dissociation of Nrf2 from its cytoplasmic anchor Kelch-like ECH-associated protein 1 (Keap1) mediates its nuclear translocation and the subsequent transcriptional upregulation of targets of the ARE pathway [16,17,18]. Among the components of this pathway, two key antioxidant enzymes, heme oxygenase-1 (HO-1) and NAD(P)H: quinone oxidoreductase (NQO1), have been shown to protect human RPE cells against oxidative stress by counteracting ROS [19,20]. HO-1, also known as heat shock protein 32 (HSP32), is involved in the degradation of heme, which leads to the production of carbon monoxide (CO), ferrous iron, and biliverdin products. The expression of HO-1, encoded by the *HMOX1* gene, is regulated at both the transcriptional and translational levels [21]. It is a pro-oxidant indicator, as it is induced by a broad range of factors such as cytokines, hormones, and growth factors; tissue damage; and exposure to blue light [22,23,24] in the retina. HO-1 is involved in various pathological processes, including oxidative stress, the inflammatory response, and ferroptosis [23]. It is an important antioxidant and a crucial component of the cellular defense system. However, studies have documented its dual nature, where the overexpression of HO-1 is linked to excessive ROS generation. Numerous recent studies have indicated the upregulation of HO-1 in disease models of AMD, including in vitro systems using blue light [25] and in vivo models employing sodium iodate [26] and light exposure [27]. Studies have also shown that high levels of free ferrous ion (Fe^2+^), a catabolic product of surplus HO-1, can cause peroxidation and oxidative stress exacerbated by ferroptosis. This occurs mainly due to the vicious cycle between the upregulation of HO-1 and oxidative stress, resulting in iron overload [23,26]. Therefore, it is imperative to maintain the ambient Nrf2/HO-1 balance for effective therapeutics.

We previously developed an oxidized low-density lipoprotein (ox-LDL)-induced in vitro human RPE injury model that involved the induction of ROS generation and lysosomal destabilization and ultimately RPE damage/death and AMD-associated pathogenesis [28]. Utilizing this model, we have identified a class of sterically hindered phenols with free radical scavenging properties that robustly protect against ox-LDL-induced RPE damage and cell death. PMC (2,2,5,7,8-pentamethyl-6-chromanol), a prototypic member of this family, also has the ability to reduce the levels of intracellular cholesterol through the NPC-1/NPC-2 efflux pathway [29]. However, the underlying molecular mechanism of PMC’s protective activity against ox-LDL-induced RPE damage is not yet fully understood and may include ROS scavenging and other direct and indirect antioxidant activities. The goal of this current study is to elucidate the antioxidant mechanism involved in PMC-mediated protection of RPE cells from ox-LDL-induced cytotoxicity.

## 2. Methods

### 2.1. The Cell Culture

Primary human fetal retinal pigmented epithelium (hRPE) cells (Lonza, Wayne, PA, USA) were cultured in 50% (*v*/*v*) DMEM/F12 (Life Technologies, Carlsbad, CA, USA)/50% (*v*/*v*) αMEM (Sigma-Aldrich, Burlington, MA, USA) supplemented with 1× penicillin–streptomycin, Glutamax, sodium pyruvate, and non-essential amino acids (all from Life Technologies, Carlsbad, CA, USA). Additionally, 10% (for growth media) or 2% (for maturation media) heat-inactivated fetal bovine serum (FBS) (Sigma-Aldrich, Burlington, MA, USA), 10 mM of nicotinamide (Sigma-Aldrich, Burlington, MA, USA), 50% N1 media supplement, 0.25 mg/mL of taurine, 0.02 μg/mL of hydrocortisone, and 0.013 ng/mL of 3,3,5-triiodo-L-thyronine (all from Sigma-Aldrich, Burlington, MA, USA) were added in accordance with the previously published protocol [30]. Half of the media was replenished every 2–3 days for 4 weeks to ensure maturation and pigment accumulation in the hRPE cells. The cells were then incubated in serum-free media supplemented overnight prior to the treatments. Human ARPE-19 cells (American Type Culture Collection, Manassas, VA, USA) were cultured in DMEM/F-12 media with L-glutamine supplemented with 1× penicillin–streptomycin and 10% FBS.

### 2.2. The Oxidized LDL (Ox-LDL) and PMC Treatments

Primary hRPE or ARPE-19 cells were cultured at a seeding density of 0.3 × 10^6^/well in 6-well plates for 2 to 4 weeks, incubated in serum-free overnight, and then treated with 200 µg/mL of oxidized LDL (ox-LDL) (cat no-770252-4, Kalen Biomedical LLC, Montgomery Village, MD, USA) with or without 1.3 µM of PMC (cat no-430676, Sigma-Aldrich, Burlington, MA, USA) for 24 and 48 h. DMSO alone (PMC diluent) was used as the control. The conditioned media were collected for analysis of the cytotoxicity, and isolation of RNA or lysate from the treatment groups was performed for subsequent studies.

### 2.3. The Cytotoxicity Assay

LDH assay (cat no-4744934001, Sigma-Aldrich, Burlington, MA, USA) was conducted to quantify cytotoxicity. Conditioned media from the cells treated with 1% Triton X-100 were used as the positive controls with the maximum LDH activity. Spontaneous LDH release was measured from the control cells without the ox-LDL and PMC treatments. The percentage cytotoxicity was estimated using the formula

% cytotoxicity = [Experimental LDH activity − spontaneous LDH activity]/[maximum LDH activity − spontaneous LDH activity] × 100.

### 2.4. Bulk RNA Sequencing

Total RNA was extracted from all treatment groups of hRPE cells using the RNeasy plus mini kit (Cat no-74034, Qiagen, Germantown, MD, USA) in accordance with the manufacturer’s instructions. The concentration and quality were determined using a NanoDrop Spectrophotometer (Thermo Fisher Scientific, Waltham, MA, USA). Bulk RNA sequencing was conducted by Azenta Life Sciences, Burlington, MA, USA, and the sequencing platform used was Illumina^@^ NovaSeq^TM^ (Azenta Life Sciences, Burlington, MA, USA). Sequence reads were trimmed using Trimmomatic V.0.26 to remove poor-quality nucleotides and possible adaptor sequences. The trimmed reads were mapped to the Homo sapiens GRCh38 reference genome using the STAR aligner v.2.5.2b. Unique gene hit counts were estimated using feature counts from the subread package v.1.5.2, following which the differential gene expression was analyzed. Wald’s test was used to generate the *p*-values and log2fold changes. Genes with an adjusted *p*-value of <0.05 and a log2fold change of >1 were termed differentially expressed genes for each comparison. A Gene Ontology analysis was conducted on the statistically significant genes using the software GeneSCF V.1.1-p2. The DESeq2 (https://genomebiology.biomedcentral.com/articles/10.1186/s13059-014-0550-8, accessed on 8 August 2025) output was used for the conditions of interest, and the GSEA (Gene Set Enrichment Analysis) approach was applied to every treatment group vs. the control independently.

Data availability: The code generated in this study was deposited into GitHub and is available at the following link: https://github.com/mcrewcow/Suman_bulkRNAseq/tree/main (accessed on 8 August 2025). The DESeq2, GSEA, and pathway-to-pattern transformation data are available on GitHub.

### 2.5. Quantitative PCR

The RNeasy plus mini kit (Cat no-74034, Qiagen, Germantown, MD, USA) was used to extract total RNA from the different treatment groups of hRPE and ARPE-19 cells, and the quality (A260/A280: ~2.0) and concentration (1 µg) of the RNA were estimated using the NanoDrop Spectrophotometer (Thermo Fisher Scientific, Waltham, MA, USA). cDNA synthesis was conducted using the SuperScript IV VILO Mastermix (cat no-11756050, Life Technologies, Carlsbad, CA, USA) as per the manufacturer’s instructions. The Techne TC-512 Thermal Cycler (Thermo Fisher Scientific, Waltham, MA, USA) was used for the annealing primer (25 °C, 10 min), reverse transcription (50 °C for 10 min), and the inactivation of the enzyme (85 °C for 5 min). Real-time qPCR was conducted using the PowerUp SYBR Green Master Mix (cat no-A25776, Thermo Fisher Scientific, Waltham, MA, USA) using LightCycler 480 (Roche, Basel, Switzerland). Thermal cycling conditions of 50 °C for 2 min, 95 °C for 2 min, and 40 cycles of 95 °C for 15 s and 60 °C for 1 min were applied in duplicates, three times for each treatment group. Amplification specificity was confirmed using the melt curve analysis. The Ct values were normalized to Gapdh (the housekeeping gene), and a comparative analysis with the control group was performed. A list of primer sequences is provided in Table 1.

### 2.6. siRNA Gene Knockdown

The hRPE cells were seeded at a density of 0.3 × 10^6^/well onto six-well plates and maintained for 2 to 4 weeks. For transfection, siRNA for *HMOX1* (cat no-sc-35554, Santa Cruz Biotechnology, Dallas, TX, USA) consisting of 3 target-specific 19–24 nt siRNAs and a scrambled siRNA control (cat no-sc-37007, Santa Cruz Biotechnology, Dallas, TX, USA) were added into the hRPE cells using lipofectamine RNAiMAX (cat no-13778075, Thermo Fisher Scientific, Waltham, MA, USA) as per the manufacturer’s instructions. Following transfection for 48 h, the scrambled siRNA- and siRNA *HMOX1*-transfected cells were treated with ox-LDL and ox-LDL along with PMC for 24 or 48 h. Conditioned media were collected from each treatment group to conduct the cytotoxicity assay for each treatment group and time point using the LDH kit (cat no-4744934001, Sigma-Aldrich, Burlington, MA, USA). Lysates were collected for an immunoblotting analysis.

### 2.7. Western Blot

Primary hRPE or ARPE-19 cells were lysed by adding 1× cell lysis buffer (cat no-9803s, Cell Signaling Technology, Danvers, MA, USA) containing a protease inhibitor (cat no-5871S, Cell Signaling Technology, USA) to the cell pellet for 20 min with occasional vortexing, followed by centrifugation at 12,000× *g* for 20 min at 4 °C. The supernatant containing the total protein was collected, and the protein concentration was determined using the BCA assay. An equal amount of protein (30 µg) was loaded onto 4–20% SDS-PAGE gel and fractionated through electrophoresis. Following this, the proteins were transferred onto a nitrocellulose membrane. The membranes were blocked in 3% bovine serum albumin for 1 h at room temperature and incubated overnight with the primary antibodies listed in Table 2. After washing with Tris-buffered saline with Tween 20 (TBST), the membrane was incubated with the secondary antibody and washed further with TBST. The fluorescent band intensity in the membranes was imaged using an infrared imaging system (Odyssey CLx Imaging System; LI-COR Biosciences, Lincoln, NE, USA). The band intensities were quantified using ImageJ 1.54g software (NIH, Bethesda, MD, USA) and graphically analyzed using GraphPad Prism (GraphPad Software Inc., Boston, MA, USA), version 10.0.3, after normalizing with the housekeeping protein (GAPDH or β-tubulin).

### 2.8. Immunocytochemistry

The ARPE-19 cells were seeded at a density of 7.5 × 10^4^ cells onto 12 mm coverslips for 2 weeks, as previously described [29]. The cells were then incubated overnight in serum-free media and treated with the ox-LDL with or without PMC for 24 to 48 h. The cells were fixed using 4% paraformaldehyde for 20 min and permeabilized using 0.1% Triton X-100 and then blocked for 1 h with 3% bovine serum albumin and incubated overnight with mouse anti-HO-1, rabbit anti-calreticulin, or mouse anti-Nrf2 antibody (Table 2). Next, the cells were incubated with Alexa Fluor 488-labeled donkey anti-rabbit (1:500, A-21206, Thermo Fisher Scientific, Waltham, MA, USA), Alexa Fluor 594-labeled donkey anti-mouse (1:500, A-21203, Thermo Fisher Scientific, Waltham, MA, USA), or Alexa Fluor 488-labeled donkey anti-mouse (1:500, A-21202, Thermo Fisher Scientific, Waltham, MA, USA) antibodies. The cells were mounted with ProLong gold antifade mounting media with DAPI (cat no-P36935, Life Technologies, Carlsbad, CA, USA) and imaged using an Axioscope 2 Mot plus microscope (Carl Zeiss Meditec AG, Zeiss, Jena, Germany). Nrf2 nuclear translocation was imaged using an SP8 confocal microscope (Leica, Wetzlar, Germany). Multiple focal planes were imaged using Z-stacking in the confocal microscope to create a three-dimensional image. For each experiment, a representative image from a minimum of six different fields was acquired. The colocalization was quantified using Manders’ coefficient via the JaCoP plugin in ImageJ 1.54g software (NIH). This coefficient measures the fraction of one fluorophore’s signal that overlaps with another, independent of the signal intensity, where a value of 0 indicates no overlap and 1 indicates complete colocalization. The fluorescent intensity was measured using ImageJ 1.54g software (NIH) in accordance with the previously published protocol [31]. Following background subtraction, the mean fluorescence intensity was calculated for each channel.

### 2.9. Reactive Oxygen Species (ROS) Detection

For ROS detection, the ARPE-19 cells were cultured on 12 mm coverslips at a density of 7.5 × 10^4^ cells for 2 weeks. The cells were incubated in serum-free media overnight and treated with the control (the DMSO diluent), ox-LDL (100 µg) with or without PMC (1.3 µM), and PMC alone for 24 h. The cells were then incubated with 1 µM of H2DCFDA (H2-DCF, DCF) (cat no-D399, Invitrogen, Carlsbad, CA, USA) for 30 min. For endoplasmic-reticulum-specific staining, the cells were incubated with 1 µM of ER-Tracker™ Red (BODIPY™ TR Glibenclamide) (cat no-E34250, Invitrogen, Carlsbad, CA, USA) for 20 min at 37 °C and then fixed with 4% paraformaldehyde for 20 min. Hoechst 33342 was used to stain the nuclei. Visualization of the cells was accomplished using the Axioscope 2 Mot plus microscope (Carl Zeiss Meditec AG, Zeiss, Jena, Germany). Colocalization quantification was performed using Manders’ coefficient in the JaCoP plugin from ImageJ 1.54g software (NIH).

### 2.10. The Statistical Analysis

The results are presented as the mean ± SEM of a minimum of three biological replicates from three independent experiments. The statistical significance was measured using a one-way ANOVA, followed by Tukey’s multiple comparison test, using GraphPad Prism version 10.0.3 between the control and treatment groups. Values of *p* < 0.05 were considered statistically significant.

## 3. Results

### 3.1. PMC Protects the RPE Against Ox-LDL-Induced Cytotoxicity

**Figure 1:**
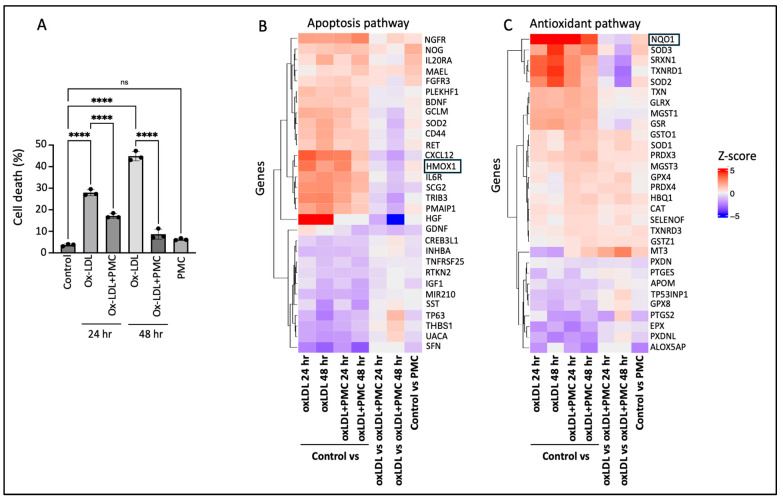
PMC protects hRPE cells from ox-LDL-induced damage by modulating apoptotic and antioxidant pathways. (**A**) hRPE cells were treated with 200 µg/mL for 24 and 48 h in the absence or presence of PMC (1.3 µM). Cytotoxicity was also measured in the untreated (control) cells and those treated with PMC alone. Cell death was measured in the condition media using the LDH assay, *n* = 3. Values are expressed as the mean ± SEM. The statistical analysis was conducted using one-way ANOVA, **** *p* < 0.0001, ns—non-significant, *n* = 3. A heatmap of the top differentially expressed genes from bulk RNA sequencing in (**B**) apoptosis and (**C**) antioxidant pathways, *n* = 3. Colors show intensity in *z*-scored units, where red shows replicates with a high expression (*z*-score = +5) and blue shows replicates with a low expression (*z*-score = −5).

Challenge with ox-LDL in the primary hRPE cells resulted in significant cell death of 27.9 ± 0.8% at 24 h and 44.9 ± 1.3% at 48 h compared to that in the control according to the LDH assay. The inclusion of PMC led to a significant (*p* < 0.0001) reduction in the cell toxicity to 17.2 ± 0.6% and 8.6 ± 1.3% at 24 and 48 h, respectively, in comparison to that in the control. PMC (1.3 µM) alone did not affect the cell viability compared to that in the control at 48 h (Figure 1A).

To gain insights into the mechanisms involved in PMC-mediated protection of the hRPE cells from ox-LDL-induced toxicity, bulk RNA sequencing was conducted. A heatmap of the differentially expressed genes between the control vs. ox-LDL, ox-LDL and PMC (24 and 48 h), PMC alone, and ox-LDL vs. ox-LDL and PMC (24 and 48 h) indicated the modulation of genes involved in the apoptosis and antioxidant pathways (Figure 1B,C). Interestingly, the antioxidant response element (ARE) genes *HMOX1* and *NQO1* were upregulated in the cells treated with ox-LDL at 24 and 48 h in comparison to those in the control. On the other hand, treatment with PMC and ox-LDL led to significantly lower levels of *HMOX1* and *NQO1* compared to those in the cells treated with ox-LDL alone at 24 and 48 h (Figure 1B,C).

### 3.2. Ox-LDL Upregulates Heme Oxygenase-1 in RPE Cells

**Figure 2:**
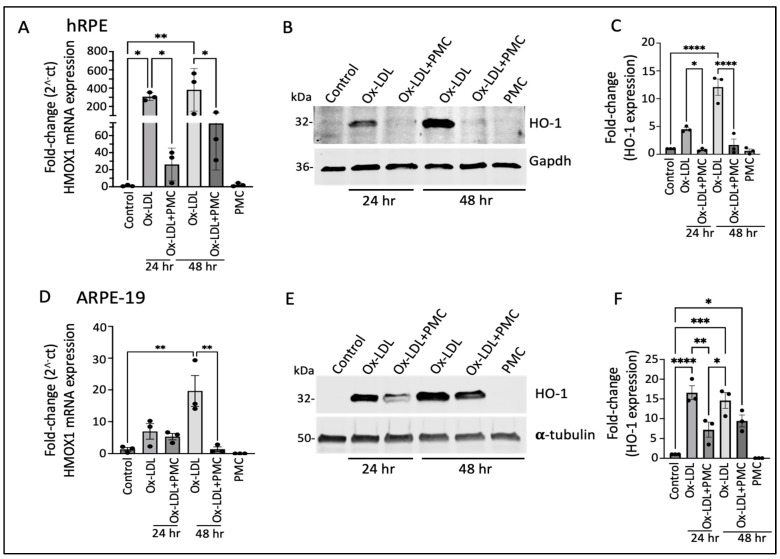
Heme oxygenase-1 upregulation in ox-LDL-treated RPE cells is suppressed by treatment with PMC. (**A**) hRPE cells matured for 4 weeks were treated with ox-LDL (200 µg/mL) with or without the presence of PMC (1.3 µM) in serum-free media. The relative *HMOX1* transcript levels were measured using qPCR. (**B**) Western blotting of the hRPE cells with the above indicated treatments at 24 and 48 h was conducted to examine HO-1 levels. (**C**) Quantification was performed using densitometry after normalization with GAPDH, *n* = 3. (**D**) HMOX-1 mRNA levels were determined through PCR in serum-starved ARPE-19 cells treated with ox-LDL in the presence or absence of PMC and qPCR. (**E**) Cell lysates from the ARPE-19 cells treated as above were examined using Western blot to determine HO-1 levels. (**F**) Quantification of the HO-1 levels was conducted after normalization with α-tubulin. *HMOX1*/HO-1 was also assessed in hRPE and ARPE-19 cells treated with PMC alone. Serum-starved untreated cells were considered as a control for all of the experiments. Values are indicated as the mean ± SEM of *n* = 3. One-way ANOVA was used for statistical analysis, * *p* < 0.05, ** *p* < 0.01, *** *p* < 0.001, **** *p* < 0.0001.

Having identified the differentially expressed genes that were potentially involved in PMC-mediated protection of the hRPE cells against ox-LDL, we validated the RNA sequencing results at the transcriptional and translational levels. The semi-quantitative qPCR analysis revealed a significant increase in *HMOX1* (encoding for the HO-1 protein) mRNA in comparison to that in the control at both 24 and 48 h upon ox-LDL challenge. Treatment with ox-LDL and PMC led to a significant reduction in the ox-LDL-induced *HMOX1* mRNA levels. At 24 h, the ox-LDL challenge led to a 307.5 ± 25.2-fold increase in *HMOX1* compared to that in the control, whereas treatment with PMC together with ox-LDL resulted in a significantly lower 26.2 ± 11.0-fold increase in *HMOX1* compared to that in the control (*p* < 0.05, ox-LDL vs. ox-LDL + PMC). At 48 h, ox-LDL alone induced a 382.3 ± 135.0-fold increase in the *HMOX1* expression compared to that in the control, while ox-LDL challenge with the PMC treatment resulted in a significantly lower increase in the *HMOX1* expression, at 74.3 ± 31.7-fold compared to that in the control (*p* < 0.05 ox-LDL vs. ox-LDL + PMC). PMC alone had no effect on the *HMOX1* levels in comparison to those in the control (Figure 2A).

Western blotting analysis of the hRPE cells revealed low expression of the HO-1 protein in the control and PMC-treated cells (Figure 2B,C). However, there was a significant increase in the levels of the HO-1 protein in the cells treated with ox-LDL at 24 and 48 h. Quantification showed a 4.5 ± 0.3-fold increase in the HO-1 levels at 24 h in comparison to those in the untreated control, which were significantly reduced 0.79 ± 0.1-fold in the cells treated with ox-LDL and PMC (*p* < 0.05, ox-LDL vs. ox-LDL + PMC). At 48 h, the HO-1 levels were significantly increased 12.1 ± 1.5-fold (*p* < 0.0001) in the cells treated with ox-LDL in comparison to those in the control, while the addition of PMC drastically suppressed the ox-LDL-induced upregulation of the HO-1 protein to 1.7 ± 1.1 (*p* < 0.0001 ox-LDL vs. ox-LDL + PMC) (Figure 2C).

Similar results were observed in the ARPE-19 cells, where there was a 7.0 ± 2.4-fold increase in the *HMOX1* mRNA levels at 24 h and a 19.6 ± 4.9-fold increase at 48 h in the cells treated with ox-LDL in comparison to those in the control. The addition of PMC reduced the *HMOX1* levels to a 5.4 ± 1.0-fold increase at 24 h. At 48 h, the addition of PMC had resulted in a 1.4 ± 0.7-fold increase in *HMOX1* levels compared to those in the untreated control, which was significantly lower compared to those under ox-LDL alone (*p* < 0.01 ox-LDL vs. ox-LDL + PMC). PMC alone had no effect on *HMOX1* mRNA expression (Figure 2D). The Western blot analysis corroborated these results. Th control cells showed low HO-1 protein expression, and PMC had no effect on HO-1 protein levels. The ox-LDL challenge resulted in the upregulation of HO-1 at 24 and 48 h, which were reduced by the addition of PMC (Figure 2E). Quantification showed that ox-LDL induced a significant increase in the HO-1 protein at 24 h (16.6 ± 1.8-fold; *p* < 0.0001) and 48 h (14.6 ± 1.9-fold; *p* < 0.001) compared to that under the control. The addition of PMC with ox-LDL at 24 h resulted in a significantly lower HO-1 level (7.2 ± 1.9-fold) in comparison to that under ox-LDL alone (*p* < 0.01, ox-LDL vs. ox-LDL + PMC), and at 48 h, the HO-1 protein levels were also trending lower (9.4 ± 1.5-fold compared to the control) compared to those under ox-LDL alone, but these data were not statistically significant (Figure 2F).

### 3.3. Ox-LDL Upregulates NQO1 in the RPE, Which Is Lowered by PMC

**Figure 3:**
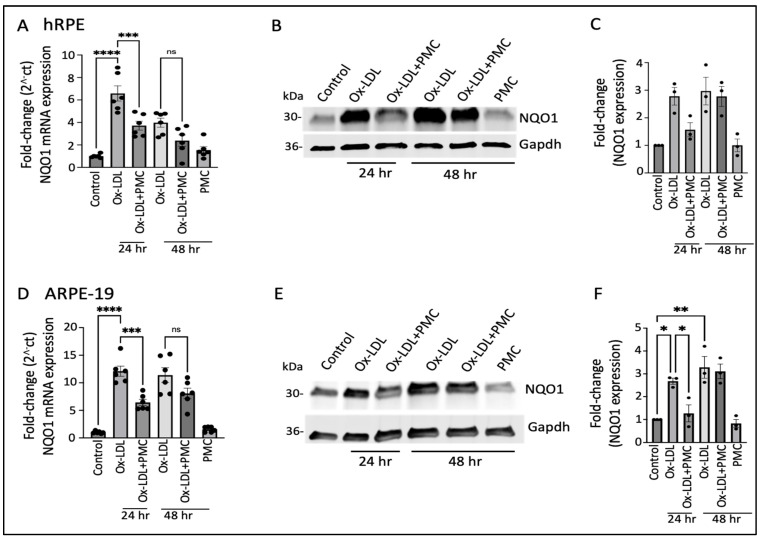
NQO1 upregulation in ox-LDL-treated RPE cells is transiently suppressed by treatment with PMC. (**A**) Serum-starved hRPE cells were treated with ox-LDL (200 µg/mL) in the presence or absence of PMC (1.3 µM). *NQO1* levels were determined using qPCR at 24 and 48 h, *n* = 6. (**B**) Western blot was conducted on cell lysates from the hRPE cells treated with ox-LDL with or without PMC to determine the NQO1 levels. Same GAPDH loading control was used for Figure 2B and Figure 3B as the targets were probed on the same membrane (**C**) Densitometry was conducted after normalization with GAPDH, *n* = 3. (**D**) Serum-starved ARPE-19 cells treated under the same experimental treatment conditions as above were analyzed for their NQO1 levels using qPCR, *n* = 6. (**E**) NQO1 protein levels were measured in cell lysates from the ARPE-19 cells in the ox-LDL-treated groups with or without PMC. (**F**) A densitometry analysis was conducted after normalization with GAPDH to quantify the NQO1 levels, *n* = 3. NQO1 levels were also measured in the hRPE and ARPE-19 cells treated with PMC alone. The serum-starved untreated cells were considered as the control for all of the experiments. Values are indicated as the mean ± SEM of the indicated *n*. A one-way ANOVA was used for the statistical analysis, * *p* < 0.05, ** *p* < 0.01, *** *p* < 0.001, **** *p* < 0.0001, ns—non-significant.

The ox-LDL treatment led to the significant upregulation of the *NQO1* mRNA levels at 24 h (6.5 ± 0.7-fold; *p* < 0.0001) and 48 h (4.0 ± 0.4-fold) in comparison to those in the controls, which were significantly reduced by PMC at 24 h (3.7 ± 0.4-fold; *p* < 0.001, ox-LDL vs. ox-LDL + PMC) and trended towards a reduction at 48 h (2.4 ± 0.5-fold) that was not statistically significant (Figure 3A). Western blot analysis indicated a similar trend in NQO1 protein expression; a 2.8 ± 0.3-fold increase in NQO1 levels was observed in the ox-LDL-treated cells at 24 h in comparison to the control levels, which were decreased 1.6 ± 0.2-fold by the addition of PMC. At 48 h, both the solely ox-LDL-treated and ox-LDL + PMC-treated cells showed similar upregulation in NQO1 levels, at 3.0 ± 0.5-fold and 2.8 ± 0.4-fold, respectively (Figure 3B,C).

Similar results were obtained using the ARPE-19 cells, with significant upregulation (12.1 ± 0.91-fold; *p* < 0.0001) in the *NQO1* mRNA at 24 h in the ox-LDL-treated cells in comparison to that in the control. The addition of PMC along with ox-LDL resulted in significantly lower levels of *NQO1* mRNA induction at 24 h (6.4 ± 0.6-fold; *p* < 0.001, ox-LDL vs. ox-LDL + PMC). At 48 h, there were 11.4 ± 1.3-fold and 8.1 ± 0.9-fold increases in the NQO1 levels in the cells treated with ox-LDL or ox-LDL plus PMC, respectively, compared to those in the controls. PMC alone had no effect on *NQO1* levels (Figure 3D). NQO1 protein levels were significantly increased at 24 h (2.7 ± 0.1-fold; *p* < 0.05) and 48 h (3.3 ± 0.5-fold; *p* < 0.01) under the ox-LDL treatments compared to those in the controls. In contrast, a significantly lower level of NQO1 (1.3 ± 0.4-fold) was observed in the ox-LDL- and PMC-treated cells at 24 h compared to that in the ox-LDL-treated cells (*p* < 0.05). At 48 h, the level of NQO1 with the ox-LDL + PMC treatment (3.1 ± 0.3-fold compared to the control) was similar to that for the ox-LDL-treated cells (Figure 3E,F).

### 3.4. PMC Reduces Ox-LDL-Induced ROS

**Figure 4:**
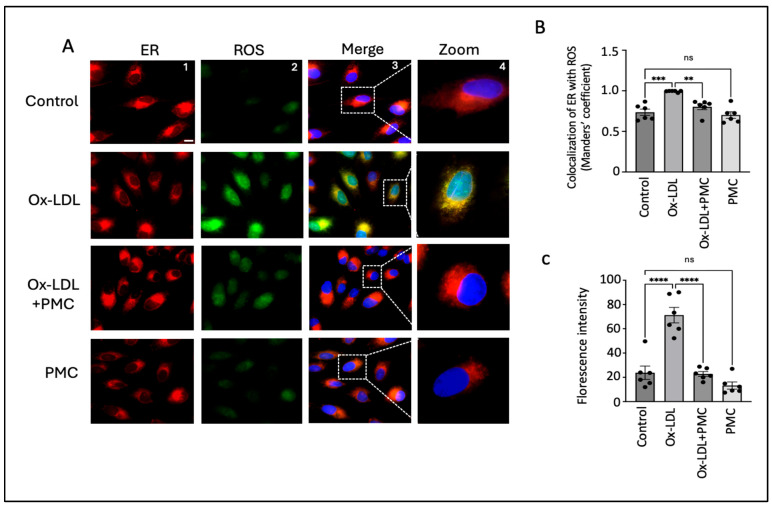
PMC prevents ox-LDL-induced oxidative stress. (**A**) ARPE-19 cells were treated with ox-LDL (200 µg/mL) in the presence/absence of PMC (1.3 µM) for 24 h. ROS levels were visualized using H2DCFDA staining (green), and the localization of ROS in the ER was estimated using the ER-Tracker™ (red). Changes in ROS levels in the ER were also examined in the cells treated with PMC. Untreated cells were considered as the controls. DAPI was used to stain the nucleus (blue); scale bar = 10 µm. Panel 4 (zoom) are higher-magnification images of the marked area in panel 3 (merge). (**B**) Quantification of colocalization of ROS in the ER was carried out using the Manders’ coefficient in the ImageJ 1.54g JaCoP plugin. (**C**) Quantification of the fluorescence intensity was conducted using ImageJ 1.54g. Values are indicated as the mean ± SEM of *n* = 6. A one-way ANOVA was used for the statistical analysis, ** *p* < 0.01, *** *p* < 0.001, **** *p* < 0.0001, ns—non-significant.

HO-1, an ER-anchored protein, is known to play an important role during ER stress [32]. Thus, to investigate changes in the intracellular ROS levels specifically in the ER, we treated ARPE-19 cells with ox-LDL, ox-LDL plus PMC, or PMC alone for 24 h and compared the changes in ROS levels to those in the control. Since the responses to ox-LDL and PMC by the ARPE19 cells were confirmed to be comparable to those for the hRPE cells in all experiments above, ARPE19 cells were used for the subsequent mechanism of action experiments. Ox-LDL treatment resulted in elevated levels of ROS that were largely localized in the ER in comparison to those in the control (Figure 4A). Quantification using the Manders’ coefficient confirmed the significant colocalization of elevated ROS in the ER in the ox-LDL-treated cells compared to that in the control (0.9; *p* < 0.001) (Figure 4B). The addition of PMC along with ox-LDL led to significantly lower levels of ROS generation, as well as ROS/ER colocalization (0.8; *p* < 0.01), compared to those in the ox-LDL-treated cells (Figure 4A,B). PMC alone had no effect on ROS levels or colocalization with the ER compared to those in the control (Figure 4A,B). Quantification using the mean fluorescence intensity supported these findings further, as elevated levels of ROS were observed in the ox-LDL-treated cells compared to those in the control (71; *p* < 0.0001). The addition of PMC with ox-LDL significantly reduced ROS levels (23; *p* < 0.0001) compared to those in the ox-LDL-treated cells. The cells treated solely with PMC did not show significantly altered ROS levels in comparison to control cells (Figure 4C).

### 3.5. Ox-LDL Stimulates Nrf2 Nuclear Translocation

**Figure 5:**
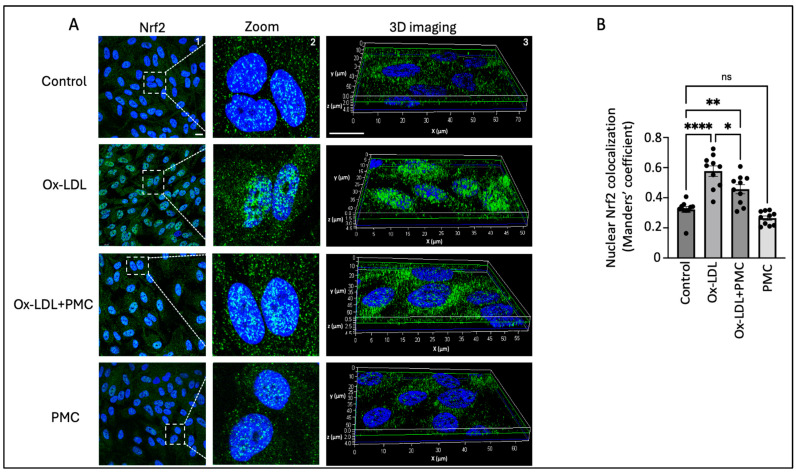
PMC prevents the nuclear translocation of Nrf2. (**A**) The nuclear translocation of Nrf2 (green) was visualized in APRE-19 cells treated with ox-LDL (200 µg/mL) in the presence/absence of PMC (1.3 µM) for 24 h. The nuclear translocation of Nrf2 was also imaged in the PMC-treated cells. Untreated cells were considered as the control. Nuclei were stained using DAPI (blue); scale bar = 10 µm. Panel 2 (zoom) are higher-magnification images of the area marked in panel 1 (Nrf2). Panel 3 shows the 3D reconstruction of the Z-stack images. (**B**) Quantification of the nuclear colocalization of Nrf2 was quantified using Manders’ coefficient in the ImageJ 1.54g JaCoP plugin. Values are indicated as the mean ± SEM of *n* = 10. A one-way ANOVA was used for the statistical analysis, * *p* < 0.05, ** *p* < 0.01, **** *p* < 0.0001, ns—non-significant.

We next investigated the ROS-mediated activation and nuclear translocation of Nrf2 because of its important role in the antioxidant response in the RPE. The results indicated the upregulation of the Nrf2 protein in the nucleus of the cells treated with ox-LDL for 24 h in comparison to that in the control. This was further confirmed through a 3D reconstruction of Z-stack confocal images of Nrf2 immunostaining (Figure 5A). Quantification of the images using the Manders’ coefficient confirmed the significant colocalization of Nrf2 in the nucleus in the ox-LDL-treated cells in comparison to that in the control (0.57 vs. 0.3; *p* < 0.0001) (Figure 5B). The cells treated with ox-LDL and PMC indicated lower levels of Nrf2 expression and a significant lower level of Nrf2 nuclear colocalization in comparison to those in the ox-LDL-treated cells (0.46 vs. 0.57; *p* < 0.05) (Figure 5A,B). PMC treatment alone caused similar levels of Nrf2 expression and nuclear colocalization to those in the control (0.27 vs. 0.3).

**Figure 6:**
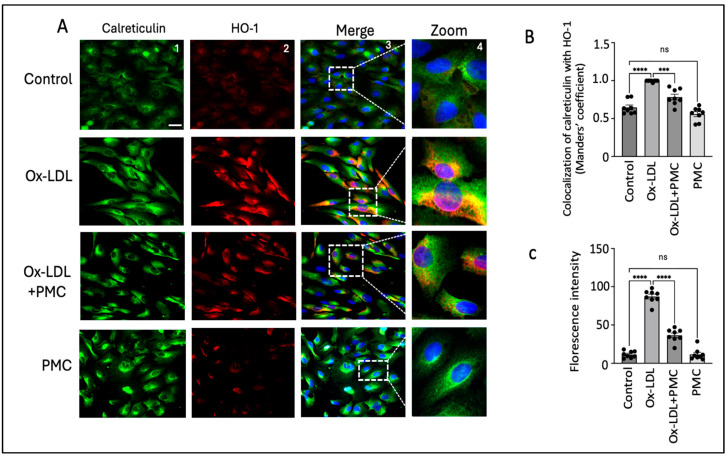
PMC prevents ox-LDL-induced HO-1 upregulation and colocalization with the ER. (**A**) ARPE-19 cells were treated with ox-LDL (200 µg/mL) with or without PMC (1.3 µM) for 24 h. Following treatment, HO-1 (red) and ER localization with calreticulin (green) were visualized. Nuclei were visualized using DAPI (blue); scale bar = 25 µm. Panel 4 (zoom) is the higher-magnification images of the area marked in panel 3. (**B**) Quantification of HO-1’s colocalization with calreticulin was quantified based on the Manders’ coefficient in the ImageJ 1.54g JaCoP plugin. (**C**) Quantification of the fluorescence intensity was conducted using Image J. Values are indicated as the mean ± SEM of *n* = 8. A one-way ANOVA was used for the statistical analysis, *** *p* < 0.001, **** *p* < 0.0001, ns—non-significant.

Nrf2 nuclear translocation leads to the activation of antioxidant response elements, which activate HO-1. Since HO-1 is localized to the ER, we visualized the effects of ox-LDL treatment with and without PMC on the HO-1 levels in the ER [33]. Using the ER marker, calreticulin, we observed high HO-1 levels mainly in the ER of the ARPE-19 cells treated with ox-LDL for 24 h in comparison to those in the control (Figure 6A). Quantification using the Manders’ coefficient confirmed the significantly elevated colocalization of HO-1 with calreticulin in the ER in comparison to that in the control (0.9 vs. 0.6; *p* < 0.0001) (Figure 6B). The cells treated with ox-LDL and PMC had significantly lower levels of HO-1 and lower colocalization of HO-1 with calreticulin in the ER in comparison to ox-LDL alone (0.8 vs. 0.9; *p* < 0.001) (Figure 6A,B). PMC had no effect on the HO-1 expression levels or its colocalization with calreticulin, which was similar to that in the control (0.5 vs. 0.6) (Figure 6A,B). Quantification via the mean fluorescence intensity supported these findings further, as the ox-LDL treatment significantly elevated the ROS levels compared to those in the controls (87; *p* < 0.0001). Simultaneous treatment of PMC with ox-LDL significantly reduced ROS levels (36; *p* < 0.0001) in comparison to those in the ox-LDL-treated cells. Cells treated with PMC alone did not exhibit significant changes in ROS levels compared to those in the control (Figure 6C).

### 3.6. PMC Protects Against Ox-LDL via the HO-1 Pathway

**Figure 7:**
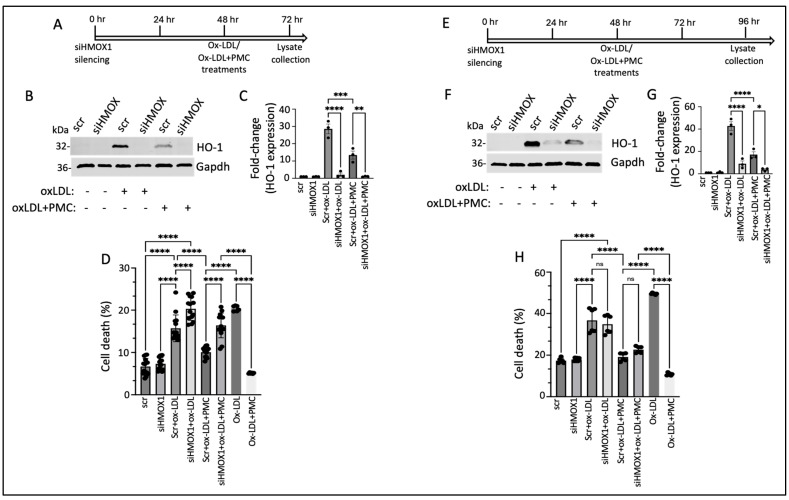
HO-1 contributes to PMC protection against ox-LDL. (**A**) An illustration showing the timeline of 48 h siHMOX1 silencing and 24 h ox-LDL treatments in the presence or absence of PMC in the hRPE cells. (**B**) Western blotting was conducted to estimate the HO-1 levels in the hRPE cells subjected to siScr or siHMOX1 for 48 h followed by ox-LDL (200 µg/mL) treatments with or without PMC (1.3 µM). (**C**) Quantification of the HO-1 levels was performed using densitometry after normalization with GAPDH, *n* = 3. (**D**) The LDH levels were measured in the conditioned media from the siScr- or siHMOX-treated cells along with the siScr and siHMOX1 cells that were treated with ox-LDL with or without PMC, *n* = 14. (**E**) An illustration of the timeline indicating 48 h siHMOX1 silencing in the hRPE cells, followed by the additional 48 h treatment with ox-LDL with/without PMC. (**F**) HO-1 levels were detected in the hRPE cells treated with siScr or siHMOX1 for 48 h, followed by ox-LDL in the presence or absence of PMC using Western blotting. (**G**) A densitometry analysis was conducted to quantify the HO-1 levels after normalization with GAPDH, *n* = 3. (**H**) Cytotoxicity was analyzed by assaying LDH in the conditioned siScr and siHMOX1 media and siScr and siHMOX cells that were subjected to ox-LDL with/without PMC treatment, *n* = 6. Cells treated with ox-LDL in the presence or absence of PMC without siScr or siHMOX1 were used as the control for both 24 h and 48 h ox-LDL and ox-LDL + PMC treatments, *n* = 6. Values are indicated as the mean ± SEM of the indicated *n*. A one-way ANOVA was used for the statistical analysis, * *p* < 0.05, ** *p* < 0.01, *** *p* < 0.001, **** *p* < 0.0001, ns—non-significant.

We next investigated the functional role of HO-1 in PMC-mediated cytoprotection against ox-LDL. This was accomplished by suppressing the expression of *HMOX1* using siRNA (siHMOX1) for 48 h, followed by treatment with ox-LDL or ox-LDL plus PMC for an additional 24 h, in the hRPE cells (Figure 7A). Conditioned media were collected for an evaluation of the cytotoxicity, and cell lysates were collected for a protein analysis. HO-1 was undetectable in the siRNA control (siScr)- and the siHMOX1-treated control cells (Figure 7B,C). The addition of ox-LDL for 24 h led to significantly elevated HO-1 levels in the siScr-treated control cells (28.6 ± 2.8-fold), which were significantly suppressed by siHMOX (2.0 ± 1.05-fold; *p* < 0.0001 compared to siScr), confirming highly efficient knockdown of the *HMOX1* expression. Treatment with ox-LDL and PMC for 24 h led to significantly lower levels of HO-1 in the siScr cells in comparison to those in the siScr cells treated with ox-LDL (13.5 ± 2.3-fold vs. 28.6 ± 2.8-fold; *p* < 0.001). The siHMOX1-silenced cells treated with ox-LDL and PMC had significantly lower levels of HO-1 in comparison to those in the siScr cells treated with ox-LDL plus PMC (1.1 ± 0.1-fold vs. 13.5 ± 2.3-fold; *p* < 0.01), further confirming *HMOX1* expression silencing at the protein level (Figure 7B,C).

A cytotoxicity analysis of the cells treated with ox-LDL without siScr or siHMOX1 displayed 20.4 ± 0.3% cell death, which was significantly reduced by PMC to 5 ± 0.1% (*p* < 0.0001). Treatment of the hRPE cells with siHMOX1 resulted in a very mildly cytotoxic effect which was comparable to that for the siScr control (7.3 ± 0.4% vs. 6.7 ± 0.5%). The introduction of ox-LDL into the siScr-treated (control) cells led to a significant increase in cell death compared to that for the siScr control (15.7 ± 0.8% vs. 6.7 ± 0.5%; *p* < 0.0001). The addition of ox-LDL to the siHMOX1-treated cells resulted in a further significant increase in cell death compared to that in the siScr cells treated with ox-LDL (20.3 ± 0.7%. vs. 15.7 ± 0.8%, *p* < 0.0001), indicating that *HMOX1* is involved in the protection of cells from ox-LDL-induced cytotoxicity. To assess the therapeutic efficacy of PMC in the presence and absence of *HMOX1,* cell death was measured in siScr cells and siHMOX1 cells treated with ox-LDL plus PMC. The results indicated 10.1 ± 0.3% cell death in the siScr cells treated with ox-LDL plus PMC, which was significantly (*p* < 0.0001) lower than that in the siScr cells treated with ox-LDL alone. Silencing of *HMOX1* in the cells treated with ox-LDL and PMC showed reduced protection of the cells at 24 h compared to that seen for the siScr cells treated with ox-LDL plus PMC (16.4 ± 0.8% vs. 10.1 ± 0.3% cell death; *p* < 0.0001) (Figure 7D), confirming the role of HO-1 in PMC-mediated protection of the RPE cells against ox-LDL. The comparison of the % cell death induced by ox-LDL under all treatment conditions showed that the suppression of *HMOX1* was associated with aggravated cell death with and without the PMC treatment (Figure 7D).

Similar studies on the effects of *HMOX1* silencing on PMC-mediated protection against ox-LDL were conducted using hRPE cells that were treated for 48 h with ox-LDL with and without PMC (Figure 7E). Similar to the hRPE cells at 24 h (Figure 7B), the siHMOX1-treated cells displayed very low levels of HO-1 at 48 h (96 h post-siRNA). The addition of ox-LDL to the siScr cells led to a robust increase in HO-1 while siHMOX1 significantly reduced the HO-1 levels in the cells under ox-LDL treatment (42.8 ± 3.9-fold vs. 9 ± 2.5-fold; *p* < 0.0001) (Figure 7F,G). Treatment of the siScr cells with ox-LDL plus PMC led to a 17.2 ± 2.6-fold increase in HO-1 levels, which was significantly (*p* < 0.0001) lower than that in the siScr cells treated with ox-LDL alone, at 42.8 ± 3.9-fold. Additionally, siHMOX1 cells treated with ox-LDL plus PMC displayed significantly lower levels of HO-1 compared to those in the siScr cells treated with ox-LDL plus PMC (3.8 ± 0.8-fold vs. 17.2 ± 2.6-fold; *p* < 0.05) (Figure 7F,G). Furthermore, at 48 h, the siHMOX1-treated cells indicated slightly higher levels of HO-1 under ox-LDL (9 ± 2.5-fold) and ox-LDL plus PMC (3.8 ± 0.8-fold) in comparison to those under 24 h treatment with ox-LDL (2.1 ± 1.1-fold) and ox-LDL plus PMC (1.1 ± 0.1-fold) (Figure 7B,C vs. Figure 7F,G), confirming that siRNA-mediated knockdown of HO-1 in the primary hRPE cells lasted up to 96 h.

The level of cell death in response to 48 h of ox-LDL treatment with and without HO-1 knockdown and with and without PMC was measured in the cells using the LDH cytotoxicity assay. Cells treated with ox-LDL with and without PMC and with and without *HMOX1* silencing were used as controls. As expected, cells treated for 48 h with PMC and ox-LDL exhibited significantly less cell death compared to that under ox-LDL alone (10.8 ± 0.3% vs. 49 ± 0.1%; *p* < 0.0001). There were similar levels of cell death in the siScr (17 ± 0.5%) and siHMOX1 (18 ± 0.3%) cells. Ox-LDL treatment of the siScr cells led to comparable levels of cell death to those for the siHMOX1 cells under ox-LDL treatment (36.7 ± 2.4% vs. 34.8 ± 1.8%) (Figure 7H). The inclusion of PMC in the ox-LDL-treated siScr cells led to significantly less cell death in comparison to that in the siScr cells treated solely with ox-LDL (19.0 ± 0.8% vs. 36.7 ± 2.4%; *p* < 0.0001). Interestingly, in contrast to the 24 h treatment, 48 h treatment with ox-LDL plus PMC in the siHMOX1 cells resulted in comparable levels of cell death to those in the siScr cells treated with ox-LDL plus PMC (22.6 ± 0.6% vs. 19.0 ± 0.8%; *p* > 0.05) (Figure 7H). This observation at 48 h further corroborated the role of HO-1 in PMC-mediated protection of the cells against ox-LDL.

### 3.7. The Continuous Presence of PMC Provides Optimal Protection to the RPE Against Ox-LDL

**Figure 8:**
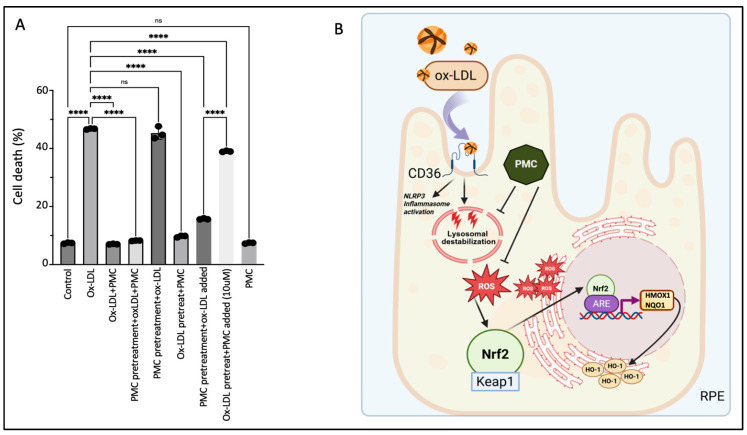
Continuous presence of PMC is required for protection against ox-LDL. (**A**) Serum-starved hRPE cells were treated with either ox-LDL (200 µg/mL) and PMC (1.3 µM) alone, ox-LDL and PMC under different treatment conditions. ox-LDL + PMC denotes simultaneous treatment, while in the other treatment groups, either the cells were pretreated with PMC or with ox-LDL. Following these treatments, the media were replaced with ox-LDL + PMC, ox-LDL alone, or PMC alone. In another group, the media were not replaced, and treatments were added to the same media. Cells were pretreated with PMC and ox-LDL was added to the same media, or they were pretreated with ox-LDL and an approximately 10-fold higher PMC concentration (10 µM) was added to the same media. Untreated cells were considered as controls. Values are indicated as the mean ± SEM of *n* = 3. A one-way ANOVA was used for the statistical analysis, **** *p* < 0.0001, ns—non-significant. (**B**) A graphical summary of PMC-mediated protection against ox-LDL in the RPE cells. Uptake of ox-LDL via the CD36 receptor [28] causes lysosomal destabilization [29] and oxidative stress in the RPE, leading to ROS generation. This triggers Nrf2 dissociation from Keap1 and its translocation to the nucleus, where it interacts with the specific promoter region, ARE, leading to the upregulation of *HMOX1* and NQO1. PMC prevents this upregulation of *HMOX1*/HO-1 and NQO1 levels by preventing ROS generation. Created using BioRender.

Concurrent treatment using PMC with ox-LDL prevented ox-LDL-induced cellular death by over 80%; ox-LDL treatment for 48 h induced significant cell death at 46.7 ± 0.1% compared to that in the untreated control cells, at 7.4 ± 0.1% (*p* < 0.0001), while simultaneous ox-LDL and PMC treatment significantly decreased the cell death to the control levels, at 7.0 ± 0.1%, compared to that with ox-LDL alone (*p* < 0.0001) (Figure 8A). Next, we investigated the effects of PMC-mediated protection under different treatment conditions and durations. Pretreatment of the hRPE cells with PMC for 24 h followed by complete replacement of the media with the ox-LDL plus PMC treatment for an additional 48 h resulted in a high level of cellular protection (8.2 ± 0.1% cell death), as seen with 48 h of simultaneous ox-LDL and PMC treatment (Figure 8A). Pretreatment of the hRPE cells with PMC for 24 h followed by complete replacement with media containing ox-LDL for an additional 48 h did not provide any cellular protection (45.3 ± 1.2% cell death), which was similar to that observed with 48 h of ox-LDL treatment alone. Exposure of the cells pretreated with PMC (24 h) and the addition of ox-LDL to the same media without a change in media led to significant protection of the cells at 48 h as compared to that seen for the ox-LDL-treated cells (15.7 ± 0.2% vs. 46.7 ± 0.1%; *p* < 0.0001). Together, these data indicated that the continuous presence of PMC provides the optimal protection to cells against ox-LDL, suggesting that PMC likely has short pharmacokinetics in RPE cells.

Pretreatment of the cells with ox-LDL for 48 h to allow for some cellular uptake of ox-LDL, followed by complete replacement with media containing PMC, provided significant cellular protection compared to that at 48 h for the ox-LDL-treated cells (9.7 ± 0.2% vs. 46.7 ± 0.1% cell death; *p* < 0.0001) (Figure 8A). However, although pretreatment with ox-LDL for 48 h followed by the addition of a high concentration of PMC at 10 µM to the same culture media provided significant protection compared to that seen for the ox-LDL-treated cells (39 ± 0.1% vs. 46.7 ± 0.1%; *p* < 0.0001), there was significantly more cell death compared to that in the PMC-pretreated cells with ox-LDL added to the same media (39 ± 0.1% vs. 15.7 ± 0.1%). These results indicated that PMC is not very effective in preventing cell death for cells that are severely damaged due to prolonged (>48 h) and continuous exposure to ox-LDL without PMC in this model. PMC treatment alone had no effect on cell viability (7.4 ± 0.1% cell death), which was similar to that in the control (7.4 ± 0.1%) (Figure 8A).

## 4. Discussion

AMD is a retinal degenerative disease characterized by the formation and accumulation of drusen [34]. Lipids, including lipofuscin and phospholipids of the phagocytized rod outer segments, are the major components of drusen [35] and are prone to oxidation. Drusen expansion and coalescence lead to RPE dysfunction/atrophy and photoreceptor cell death, which are the hallmarks of AMD-associated pathologies [36,37]. Numerous studies have confirmed the presence of significantly higher levels of oxidized phospholipids in the macula [9] and plasma [38] of AMD patients, supporting the correlation between oxidized lipid exposure and AMD pathogenesis.

Previous studies in our lab have shown uptake of ox-LDL through the CD36 receptor and the dose-dependent cytotoxicity of ox-LDL in RPE cells [28]. In line with this, our lab reported the significant role of NLRP3 inflammasome activation as a major contributor to cytokine release and cell death in ox-LDL-treated RPE cells [28]. Based on these observations, ox-LDL-induced damage to RPE cells was used as a model in the present study to mimic AMD-associated pathologies in RPE cells to determine the mechanism of PMC-mediated protection of the RPE against ox-LDL. Using this model, our study unraveled the key antioxidant pathway through which PMC protects the RPE cells from the oxidative damage and cytotoxicity induced by ox-LDL. We show the efficacy of PMC in preventing ox-LD-induced ROS generation, thus preventing activation of the Nrf2-ARE-*HMOX1*/NQO1 pathway, which is activated during ox-LDL-induced oxidative damage.

We previously screened multiple antioxidants for their protection against ox-LDL-induced cytotoxicity. Our results revealed the significant efficacy of sterically hindered phenol compounds in protecting RPE cells from oxidative stress by scavenging free radicals and preventing ox-LDL-induced lysosomal destabilization and cell death [29]. Consistent with this, a sterically hindered phenol, PMC, presented high antioxidant and protective efficacy in RPE cells against ox-LDL and was thus used in our present study.

One of the primary pathways employed by the RPE to maintain cellular homeostasis and neutralize oxidative stress is the Nrf2 pathway [17]. Nrf2, a master transcriptional factor under oxidative stimuli and ROS generation, dissociates from Keap1 and translocates to the nucleus. The subsequent signaling cascade causes Nrf2 nuclear translocation, and interaction with the specific ARE promoter region leading to the upregulation of antioxidant genes, including *HMOX1* and NQO1 [21,39]. Additionally, HO-1 proteins that are located in the endoplasmic reticulum (ER) are also released from the ER into the cytosol under oxidative stress [21].

Due to the antioxidant and anti-inflammatory properties of HO-1, it was considered as a promising therapeutic strategy against age-related diseases of the eye. However, emerging evidence also suggests the deleterious functions of HO-1 since the overexpression of HO-1 has been linked with excessive Fe^2+^ and ROS generation, which can lead to lipid peroxidation and ferroptosis, an iron-dependent cell death pathway which is a critical hallmark of AMD [23]. Previously, one study showed that HO-1 upregulation in a NaIO_3_-induced AMD mouse model could lead to elevated iron levels, which in turn cause free-radical-mediated damage [26]. Interestingly, knockdown of HO-1 using siRNA reduced oxidative-stress-induced ferroptosis in NaIO_3_-treated ARPE-19 cells. Moreover, the suppression of HO-1 levels using a known inhibitor, Zn-protoporphyrin (ZnPP), rescued RPE degeneration and restored visual function and retinal structure in the NaIO_3_ AMD mice model [26]. In another study, knockdown of HO-1 demonstrated reduced proliferation and migration in endothelial cells, suggesting it to be a potential target in treating the choroidal neovascularization that occurs in advanced neovascular or wet AMD [40].

Previous oxidative stress models of AMD have reported similar upregulation in HO-1 to that observed in our study. Cigarette smoke extract, an important environmental risk factor for AMD, caused HO-1 upregulation and modest involvement of Nrf2 [19]. A H_2_O_2_-induced ROS production model of AMD confirmed the upregulation of HO-1 and NQO1 in human ARPE-19 cells [20]. These observations are in line with the HO-1 upregulation observed in our ox-LDL-treated cells.

Interestingly, concurrent treatment of ox-LDL with PMC resulted in lower levels of HO-1 and NQO1 in both the hRPE and ARPE-19 cells. One of the major contributors to this could be the role of PMC in preventing the upstream oxidative damage caused by internalized ox-LDL in the cells [28], which prevents Nrf2 nuclear translocation and the subsequent upregulation of HO-1 and NQO1. Notably, the transcriptional and translational changes for HO-1 and NQO1 were comparable in both the hRPE and ARPE-19 cells, validating ARPE-19 as a useful model for understanding the cellular mechanisms involved in PMC-mediated protection from ox-LDL-induced pathologies. The relevance of ARPE-19 cells as a model for the discovery of innovative therapeutic interventions has been witnessed previously [41,42].

NQO1 upregulation in response to oxidative stress via the Nrf2 signaling pathway is well established [43]. In our study, co-treatment with ox-LDL and PMC resulted in reduced NQO1 levels at 24 h compared to those under ox-LDL treatment alone, demonstrating PMC’s initial efficacy in mitigating oxidative stress. However, this inhibitory effect was not sustained at 48 h, suggesting the activation of compensatory mechanisms that restore redox homeostasis and override the early suppressive impact of PMC. Such adaptive responses have been previously reported, whereby prolonged oxidative stress induces feedback pathways to normalize antioxidant enzyme expression, including NQO1, thereby maintaining cellular redox balance [44,45].

The over-activation of HO-1 has previously been linked to retinal degeneration through ER stress. It was previously observed that low levels of HO-1 provided protection against light-induced photoreceptor cell death while high levels of HO-1 induced photoreceptor cell death and retinal degeneration [46]. Consistent with this, we observed significantly higher levels of HO-1 under the ox-LDL treatments in comparison to those under concurrent ox-LDL and PMC treatment. Based on this, we validated the efficacy of PMC in preventing ROS and the subsequent activation of the Nrf2-ARE-*HMOX1* axis. Notably, our data indicated that the suppression of *HMOX1* expression by siRNA resulted in exacerbated ox-LDL-induced cell death. Importantly, this effect was also observed in cells concurrently treated with ox-LDL and PMC, indicating that cellular protection depends on the maintenance of optimal HO-1 levels. The complete absence of HO-1 hinders the intrinsic cellular defense against oxidative stress. Our data also showed that PMC provides protection from ox-LDL and at least partly involves the Nrf2-ARE-*HMOX1* pathway since the protection by PMC against ox-LDL in the RPE cells was significantly reduced upon the suppression of *HMOX1* expression using siRNA.

While there are a number of ongoing research efforts into antioxidant therapies for AMD [37,47,48,49], sterically hindered phenols could be an attractive candidate due to their stability and ability to protect the retinal cells from the oxidative stress induced by ox-LDL, a key driver of AMD pathology. Future investigations in our lab will focus on identifying and validating multiple different pathways involved in PMC-mediated protection and defining the role of additional signaling cascades that may contribute to its therapeutic potential.

## Figures and Tables

**Table 1:** Primer sequences.

Gene Name	Forward Sequence	Reverse Sequence	Gene Accession Number
*HMOX1*	CAACATCCAGCTCTTTGAGG	GGCAGAATCTTGCACTTTG	NM_002133.3
*NQO1*	CCTGCCATTCTGAAAGGCTGGT	GTGGTGATGGAAAGCACTGCCT	NM_000903.3
*Gapdh*	GAAGGTGAAGGTCGGAGTC	GAAGATGGTGATGGGATTC	NM_001256799.3

**Table 2:** List of antibodies.

Antibody	Host Species *	Species Reactivity *	Company	Catalogue Number	Dilution **
HO-1	m	b, h, m, r, d	Thermo Fisher Scientific, Waltham, MA, USA	MA1-112	WB: 1:1000 ICC: 1:200
NQO1	m	h	Abcam, Waltham, MA, USA	ab28947	WB: 1:500
Calreticulin	Rb	h, m, r	Cell Signaling Technology, Danvers, MA, USA	D3E6	ICC: 1:200
Nrf2	m	h	Santa Cruz Biotechnology, Dallas, TX, USA	sc-365949	WB: 1:500 ICC: 1:100
GAPDH	Rb	h, m, r, mk	Cell Signaling Technology, Danvers, MA, USA	D16H11	WB: 1:1000
α-tubulin	Rb	h, m, r, mk, b	Cell Signaling Technology, Danvers, MA, USA	2144	WB: 1:1000

* m—mouse; Rb—rabbit; h—human; r—rat; b—bovine; d—dog; mk—monkey. ** WB—Western blotting; ICC—immunocytochemistry.

## Data Availability

The datasets and materials used in this study are available upon request from the corresponding author.
